# Inflammatory and metabolic markers mediate the association between urinary metals and non-alcoholic fatty liver disease in U.S. adults: a cross-sectional study

**DOI:** 10.3389/fpubh.2025.1564302

**Published:** 2025-07-04

**Authors:** Shu-yue Li, Jia-jie Lv, Xi-tao Yang, Cheng-hao Yang, Min-yi Yin

**Affiliations:** ^1^Department of Obstetrics and Gynecology, Obstetrics and Gynecology Hospital, Fudan University, Shanghai, China; ^2^Department of Vascular Surgery, Shanghai Ninth People's Hospital, Shanghai Jiao Tong University, Shanghai, China; ^3^Department of Vascular Surgery, Shanghai Putuo People's Hospital, School of Medicine, Tongji University, Shanghai, China

**Keywords:** heavy metals, non-alcoholic fatty liver disease, inflammatory markers, WQS regression, mediating effect

## Abstract

**Background:**

Non-alcoholic fatty liver disease (NAFLD) is a global public health problem. Inflammation, oxidative stress, and insulin resistance are involved in the development and progression of NAFLD. Although the etiology of NAFLD remains unclear, environmental factors are increasingly recognized as non-negligible risk factors. This study was to evaluate the urine metal associated with the risk of NAFLD and inflammation and metabolic markers mediating role.

**Methods:**

According to the national health and nutrition examination survey (NHANES), to detect the metal concentration in the urine of 3,948 U.S. adults, including barium (Ba), cadmium (Cd), cobalt (Co), and cesium (Cs), molybdenum (Mo), lead (Pb), antimony (Sb), thallium (Tl), and uranium (Tu). Multivariate logistic regression and weighted (WQS) and quantile regression were used to investigate the single and mixed metals associated with the risk of NAFLD. In addition, inflammatory and metabolic markers may mediate the relationship between metals and NAFLD. Inflammatory markers included neutrophil albumin ratio (NPAR) and neutrophil-to-lymphocyte ratio (NLR). The fatty liver index (FLI) was used as a liver metabolic marker. Mediation analysis aimed to investigate the mediating effects of inflammation and metabolism on the association between metals and NAFLD risk.

**Results:**

In the single-exposure model, Ba, Cd, Cs, Mo, Tl, and Tu were identified to be positively associated with NAFLD risk, with odds ratios (OR) ranging from 1.29 to 1.48 (all *P* < 0.05). Mixed exposure analysis showed consistent associations (OR: 1.48, 95% CI: 1.06 to 2.06). In addition, Ba, Cd, Mo, Pb, and Tu and negatively correlated with inflammatory markers, but was positively correlated with hepatic metabolism markers. At the same time we have found that inflammatory markers and negative correlation with NAFLD, and hepatic metabolism markers are positively correlated with NAFLD risk relationship (*P* < 0.05). Further mediation analysis showed that the associations of single metals (mainly Mo, Ba, and Tu) and mixed metals with NAFLD risk were mediated in parallel by the above-mentioned inflammatory and metabolic markers, with the mediating proportions ranging from 16.89% to 69.39% (all *P* < 0.05). Show that metal concentration can reduce serum inflammatory markers in the urine and raise levels of metabolites markers and then induce NAFLD.

**Conclusion:**

These findings suggest that exposure to the metal can increase the risk of NAFLD, this may be partly mediated by inflammation and metabolic markers. Clinically, this highlights the importance of monitoring environmental metal exposure and addressing inflammation and metabolic dysfunction as potential intervention targets to reduce NAFLD risk.

## Introduction

Non-alcoholic fatty liver disease (NAFLD) impacts 30.05% of the global population and is a leading cause of cirrhosis and hepatocellular carcinoma (HCC) (1, 2). Its manifestations range from simple steatosis to the more serious non-alcoholic steatohepatitis (NASH) (1, 3). In the U.S., prevalence rates are about 34% or higher, with 3%−5% of patients experiencing progressive NASH (4). Recent meta-analyses have shown a significant rise in NAFLD cases in Asia, currently estimated at 29.6%, with notable variations between countries (5). NAFLD can progress to liver cirrhosis and increase the risk of HCC, with patients facing heightened mortality risks from liver disease, cardiovascular issues, and cancers (1, 6). The challenge in predicting NAFLD outcomes lies in the yet-to-be-identified factors driving its progression (7).

Heavy metals, ubiquitous in the environment including air, soil, water, and food, pose significant health hazards (8). Their exposure has been linked to diseases such as diabetes and cancer, marking them as a major global public health issue (9, 10). Epidemiological studies suggest a positive correlation between the prevalence of NAFLD and levels of arsenic, lead, mercury, cadmium, and manganese (11, 12). Recent studies further support the association between heavy metal exposure and NAFLD in different populations, including adolescents and adults. For example, Lee et al. (13) reported a link between heavy metals and biomarkers of NAFLD in Korean adolescents. Similarly, Xie et al. (14) demonstrated associations of metal mixtures with metabolic-associated fatty liver disease and NAFLD using NHANES data. Zhang et al. (15) further reinforced these findings by identifying a significant association between urinary nickel levels and the risk of NAFLD and liver fibrosis in a U.S. adult population, with the effect being more pronounced in men. Toxicological research further reveals that heavy metals, along with dioxins and polychlorinated biphenyls (PCBs), play a role in NAFLD onset (12, 16). Most studies have investigated the impact of individual metals on NAFLD, but real-world exposure often involves multiple metals, which can have combined effects—synergistic, antagonistic, or otherwise—distinct from single metal exposure (9, 11, 12, 16).

Systemic inflammation and liver metabolism are integral to the development of NAFLD and advanced cirrhosis (17). Biomarkers like the neutrophil-to-albumin ratio (NPAR) and neutrophil-to-lymphocyte ratio (NLR) are crucial in this context (18). NPAR, calculated using neutrophil counts and albumin levels, provides a cost-effective and accessible measure of systemic inflammation (19, 20). NLR, derived from neutrophil and lymphocyte counts, is easily obtained from routine blood tests (21). Both NPAR and NLR are effective in evaluating the severity of NAFLD and liver fibrosis (18–21). NPAR has proven useful in predicting conditions such as acute kidney injury, cardiogenic shock, myocardial infarction, and cancer. Additionally, the fatty liver index (FLI), a non-invasive metric combining waist circumference, body mass index, triglyceride levels, and glutamine transaminase, aids in fatty liver diagnosis through ultrasound (18, 22).

Considering the impact of inflammation and metabolic markers in relation to metal exposure and NAFLD, we hypothesize that heavy metal exposure may intensify NAFLD risk by affecting these markers. To test this hypothesis, we conducted a cross-sectional study using data from 2013 to 2020 national health and nutrition examination survey (NHANES), investigating the link between nine urinary metals and NAFLD risk. Urinary metals were selected for this study because they reflect recent exposure and are non-invasive to collect, making them practical for large population studies (23). Furthermore, urinary metal concentrations are considered reliable biomarkers of internal dose and offer insight into the body's excretion and detoxification processes. This study also examines the mediating roles of various inflammatory and metabolic markers in this association.

## Methods

### Study population

**Figure 1:**
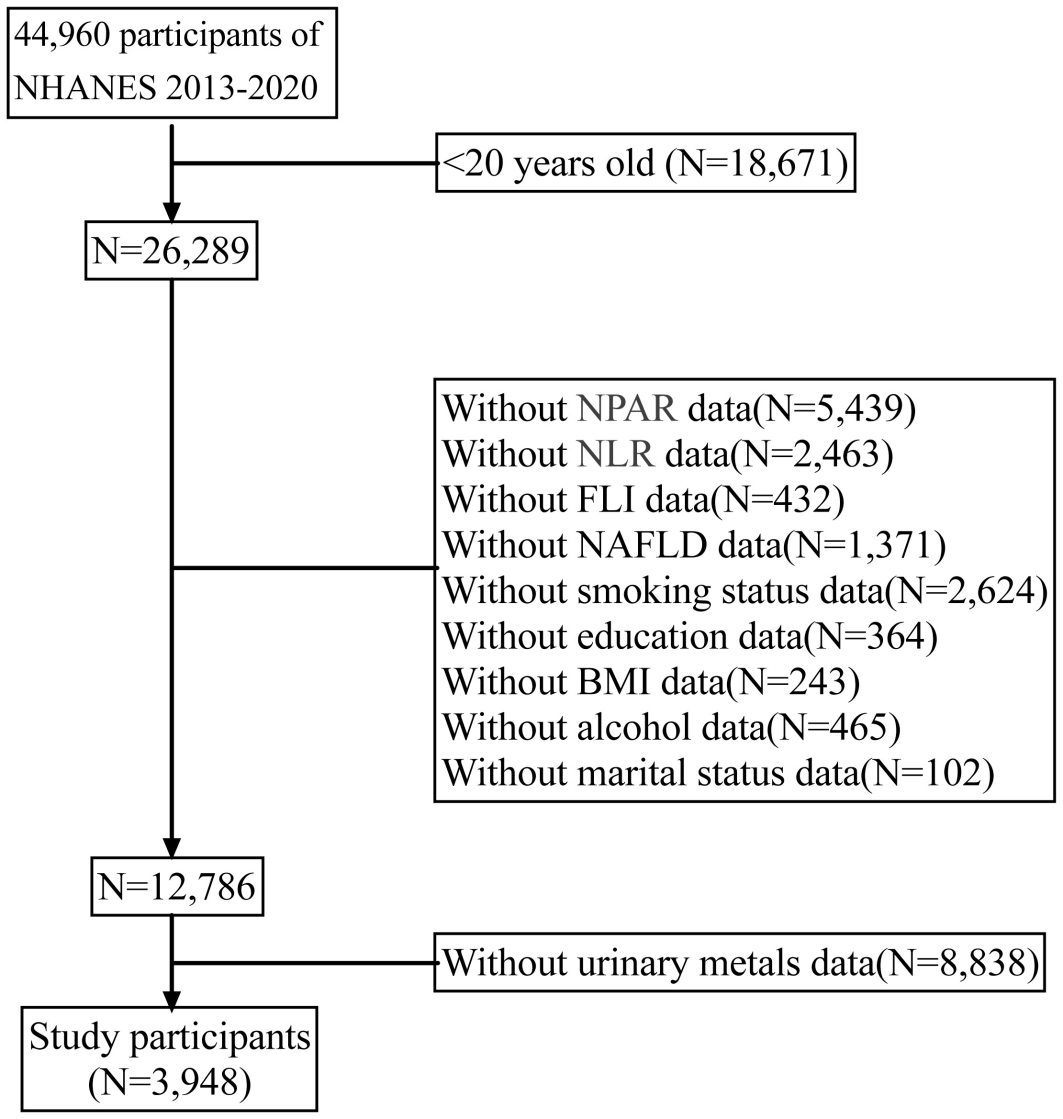
Flow chart of the study.

The national health and nutrition examination survey (NHANES) is a comprehensive, interdisciplinary survey program initiated by the centers for disease control and prevention (CDC) to evaluate the health and nutrition status of U.S. residents. The overarching objective of NHANES is to gather, scrutinize, and publish data on the health, nutrition, and environmental exposures of U.S. residents. NHANES has been administered annually since the 1960s and encompasses individuals of all ages across the United States. For the present analysis, we amalgamated four survey periods (i.e., 2013–2014, 2015–2016, 2017–2018, and 2019–2020) to generate estimates with heightened precision and less sampling error. In the current study, NAFLD patients were included according to assessment (*n* = 44,960). Next, we excluded individuals whose information on nine metals was missing (*n* = 8,838). Collectively, 3,948 participants were enrolled (Figure 1).

### Statement

The work has been reported in line with theSTROCSS criteria (24).

### NAFLD assessment

Liver stiffness was assessed using the controlled attenuation parameter (CAP) of the Fibroscan model (Echosens North America, Waltham, MA, USA), a reliable indicator of liver fibrosis. Liver steatosis, with an AUROC of 0.96, was identified through the non-invasive vibration controlled transient elastography (VCTE) method (25). Within the NHANES dataset, 4,266 individuals underwent VCTE assessments using the FibroScan model 502 V2 Touch (Echosens, North America) equipped with either a medium (M) or extra-large (XL) wand in the NHANES mobile examination centers (MEC). Consistent with prior research, NAFLD was categorized as having a CAP value of 285 dB/m or higher (26).

### Metal measurement

Data on nine urinary metals from the NHANES 2013–2020 dataset were analyzed. These metals, including barium (Ba), cadmium (Cd), cobalt (Co), cesium (Cs), molybdenum (Mo), lead (Pb), antimony (Sb), thallium (Tl), and tungsten (Tu), were detected in spot urine samples using inductively coupled plasma mass spectrometry (ICP-MS). For values below the detection limit, the limit of detection (LOD) divided by the square root of two was employed as a substitute. The protocols for quality assurance and control in NHANES adhere to the requirements set by the 1988 Clinical Laboratory Improvement Act.

### Measurement of inflammatory and metabolic markers

Hematological parameters in the NHANES CBC Profile were analyzed using the Beckman Coulter Automated Hematology Analyzer DxH 900 (Beckman-Coulter, Brea, CA, USA). This system performs comprehensive blood analyses, including counts of red and white cells, hemoglobin, hematocrit, and red blood cell indices, utilizing an automatic dilution and mixing system for sample processing and a single beam photometer for hemoglobin measurement. The Coulter VCS system conducts the WBC differential. The neutrophil-to-lymphocyte ratio (NLR) was calculated by dividing each participant's absolute neutrophil count by their absolute lymphocyte count. The neutrophil-to-albumin ratio (NPAR) was derived using the formula: Neutrophil percentage (of total WBC count) × 100/Albumin (g/dL). Moreover, venous blood samples were collected post an overnight fast. The presence of serum HBV surface antigen (HBsAg) was assessed using a radioimmunoassay, and the antibody to HCV (anti-HCV) was detected through a second-generation enzyme immunoassay, both performed by Abbott Laboratories, North Chicago, IL, USA. The Roche/Hitachi Modular Analytics System (Roche Diagnostics GmbH, Mannheim, Germany) was employed for measuring serum biochemical markers. The fatty liver index (FLI) was computed using the formula: FLI = (e^0.953^ ln (TG) + 0.139 BMI + 0.718 ln (GGT) + 0.053 WC – 15.745) / (1 + e^0.953^ ln (TG) + 0.139 BMI + 0.718 ln (GGT) + 0.053 WC – 15.745) ^*^ 100. The liver adipose pathology (LAP) score was calculated as follows: LAP = (waist circumference (cm) – 58) × triglycerides (mmol/L).

### Covariates

Based on previous research and clinical experience, the sociodemographic characteristics considered in this study included age, sex, race (Mexican American, White, Black, etc.), education level (below high school, high school, and beyond), marital status (living with a partner, single, married), and poverty income ratio. The researchers also evaluated the poverty income ratio (PIR), smoking status (former smoker, never smoker, and current smoker), alcohol consumption status (never smoker, former smoker, light, moderate, and heavy), body mass index (BMI), metabolic equivalent (MET), and kidney failure (16, 18). Participants who had never smoked or had smoked fewer than 100 cigarettes in their lifetime were classified as never smokers; participants who reported smoking 100 cigarettes in their lifetime but did not currently smoke were classified as former smokers; current smokers were defined as those who smoked 100 cigarettes a day or on any given day. PIR is a measure of socioeconomic status that compares total household income to the poverty line. It is divided into three categories: low (PIR < 1.35), medium (1.35 ≤ PIR < 3.0), and high (PIR ≥ 3.0). Regarding alcohol consumption, people who reported having fewer than 12 drinks were defined as never drinkers, while former drinkers were those who reported having more than 12 drinks in their lifetime but had not drunk in the previous year; current drinkers were further classified as light, moderate, and heavy drinkers. Binge drinking was defined as three or more drinks a day for women, four or more drinks a day for men, or five or more drinks a month. Moderate drinkers were defined as having two drinks a day for women and three drinks a day for men and binge drinking twice or less a month. Comorbidities such as diabetes, congestive heart failure, coronary artery disease, chronic obstructive pulmonary disease (chronic bronchitis and/or emphysema), hypertension, and cancer were also incorporated. Each disease was scored on a scale ranging from 1 to 6, with higher scores reflecting a greater impact on a patient's health status and prognosis. The comorbidity index score could be calculated by summing up the scores for each disease, and higher CCI scores indicated more severe cases of multiple diseases.

### Statistical analysis

Participant demographics and NAFLD conditions were analyzed using chi-square and *t*-tests. To achieve normal distribution, metal concentrations were natural log-transformed for continuous variables and categorized into four quartiles (Q1–Q4) for categorical analysis. Odds ratios (ORs) and 95% confidence intervals (CIs) for the relationship between urinary metals, inflammatory metabolic markers, and NAFLD risk were estimated using multivariate logistic regression. Similarly, multivariate linear regression explored the association between metals and biological aging markers, adjusting for factors like age, gender, race/ethnicity, education, marital status, metabolic equivalent (MET), alcohol consumption, body mass index (BMI), and poverty income ratio (PIR). Pearson correlation was employed to assess the relationship between log-transformed metals. The weighted quantile sum (WQS) regression, calculated using the R package “gWQS”, assessed the cumulative impact of metal exposure on NAFLD. This approach, producing a WQS index ranging from 0 to 1, evaluates mixed exposure levels and highlights significant components. The index's outcome indicates the combined metal influence on NAFLD risk.

Initially, weighted multiple logistic regression analyzed the association between urinary metal and NAFLD, constructing three models: a crude model without adjustment, Model 1 adjusting for age, sex, and race, and Model 2, which further includes demographic factors (race, BMI, PIR, MET, education), Charlson Comorbidity Index (CCI), and lifestyle factors (smoking status, renal failure, alcohol consumption). Secondly, Bayesian Kernel Machine Regression (BKMR) assessed the combined effect of metals and the dose-response relationship of single metals to NAFLD, adjusted for other metal concentrations. Then, parallel mediation models estimated the mediating effect of biological aging markers on the association of single and mixed metals with NAFLD risk. These models use individual indicators as mediators, contrasting with serial mediation models that use paths. Mediation analysis, employing Monte Carlo methods with quasi-Bayesian approximation, simulated 5,000 times to differentiate direct (DE) and indirect effects (IE) of metal exposure on NAFLD. The mediation proportion was calculated as IE/TE (total effect). Lastly, a penalty spline method was applied for smooth curve fitting to explore the non-linear relationship between urinary metal levels and NAFLD. All statistical analyses were performed using R software version 4.3.0 (Core Team, Vienna, Austria). A *P*-value < 0.05 was considered statistically significant.

## Results

### Characteristics of participants and metals distribution

In our study of 3,948 adults, 1,435 were identified as having NAFLD. Table 1 presents demographic characteristics of participants, comparing those with and without NAFLD. Significant statistical differences were observed in several variables between NAFLD and non-NAFLD groups, including age, gender, marital status, education level, drinking habits, MET, CCI, BMI, NPAR, and FLI. The distribution of metal concentrations among the participants is detailed in Supplementary Table 1, where we found a 100% detection rate for metals. Pearson correlation analysis of Ln-transformed metals indicated a moderate correlation between Cs and Tl, with a coefficient of 0.63. Correlations among other metals were relatively weaker, as shown in Supplementary Figure 1.

**Table 1:** Characteristics of participants by NAFLD, NHANES 2011–2020.

**Variable**	**Total**	**Non-NAFLD**	**NAFLD**	***P***
**Age**, ***n*** **(%)**				<0.0001
20–42	1,334 (33.79)	1,009 (43.33)	325 (27.23)	
43–	1,373 (34.78)	858 (38.76)	515 (37.17)	
62–80	1,241 (31.43)	646 (17.91)	595 (35.59)	
**Gender**, ***n*** **(%)**				<0.001
Female	1,978 (50.1)	1,157 (47.27)	821 (56.77)	
Male	1,970 (49.9)	1,356 (52.73)	614 (43.23)	
**Race/ethnicity**, ***n*** **(%)**				0.69
Mexican American	521 (13.2)	321 (8.99)	200 (10.25)	
Non-Hispanic Black	974 (24.67)	623 (11.07)	351 (10.69)	
Non-Hispanic White	1,320 (33.43)	834 (61.98)	486 (61.07)	
Other Hispanic	394 (9.98)	263 (7.77)	131 (6.98)	
Other race/ethnicity	739 (18.72)	472 (10.18)	267 (11.02)	
**Marital status**, ***n*** **(%)**				0.01
Married/cohabiting	2,334 (59.12)	1,479 (63.11)	855 (66.52)	
Widowed/divorced/separated	898 (22.75)	530 (18.65)	368 (20.58)	
Never married	716 (18.14)	504 (18.24)	212 (12.90)	
**Education level**, ***n*** **(%)**				0.05
Under high school	761 (19.28)	445 (11.08)	316 (12.55)	
High school or equivalent	2,173 (55.04)	1,422 (61.23)	751 (56.09)	
Above high school	1,014 (25.68)	646 (27.69)	368 (31.36)	
**Alcohol intake, n (%)**				<0.0001
Heavy	709 (17.96)	562 (24.12)	147 (11.36)	
Mild	2,277 (57.67)	1,340 (53.14)	937 (64.66)	
Moderate	625 (15.83)	452 (19.07)	173 (13.69)	
Never	337 (8.54)	159 (3.66)	178 (10.28)	
**PIR**, ***n*** **(%)**				0.39
<1.3	979 (28.67)	581 (18.89)	398 (21.82)	
1.3–3.5	1,318 (38.59)	861 (34.89)	457 (34.33)	
> 3.5	1,118 (32.74)	725 (46.22)	393 (43.85)	
**Smoking status**, ***n*** **(%)**				0.29
Former	974 (24.67)	585 (25.91)	389 (29.42)	
Never	2,253 (57.07)	1,422 (57.66)	831 (55.71)	
Now	721 (18.26)	506 (16.43)	215 (14.88)	
CCI (SEx)	0.85 (0.04)	0.74 (0.05)	1.08 (0.05)	<0.0001
BMI (SEx)	29.94 (0.19)	28.94 (0.24)	32.00 (0.31)	<0.0001
MET (SEx)	6,025.26 (308.98)	6,632.93 (387.98)	4,745.68 (265.89)	<0.0001
NPAR (SEx)	1.05 (0.01)	1.03 (0.02)	1.09 (0.02)	0.01
NLR (SEx)	2.16 (0.03)	2.16 (0.03)	2.14 (0.04)	0.65
FLI (SEx)	51.53 (1.19)	47.16 (1.39)	60.26 (2.20)	<0.0001

^a^ Values are standard error (SEx) for continuous variables and weighted percentage for categorical variables; *P* values were derived from Student's *t*-test for continuous variables and the Chi-square test for categorical variables.

^b^ PIR, poverty to income ratio; BMI, body mass index; CCI, co-morbidity index; MET, metabolic equivalent; NPAR, neutrophil-to-albumin ratio; NLR, neutrophil-to-lymphocyte ratio; FLI, fatty liver index.

### Associations between metal concentration and NAFLD risk

**Figure 2:**
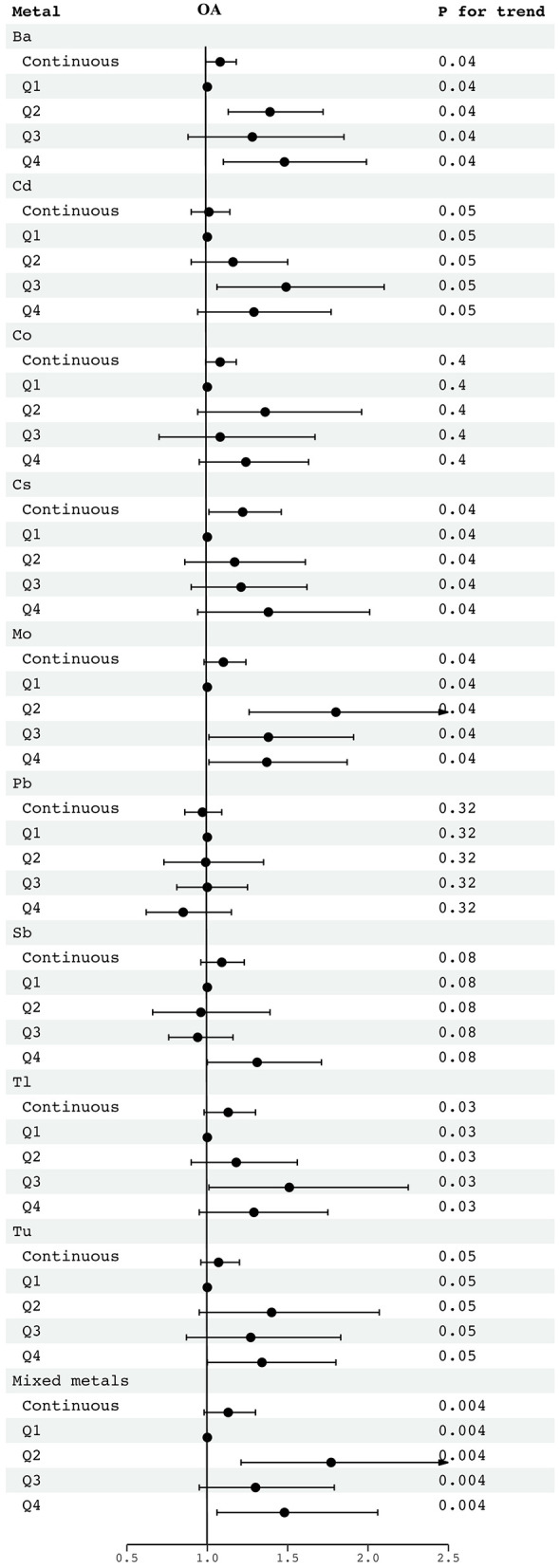
OR (95% CI) in NAFLD associated with single and mixed metals levels. Models were adjusted for gender, age, race, education, PIR, marital status, BMI, MET, drinking alcohol status, smoking status and CCI. Continuous, Ln-transformed concentration of metals; Q, quartile.

**Figure 3:**
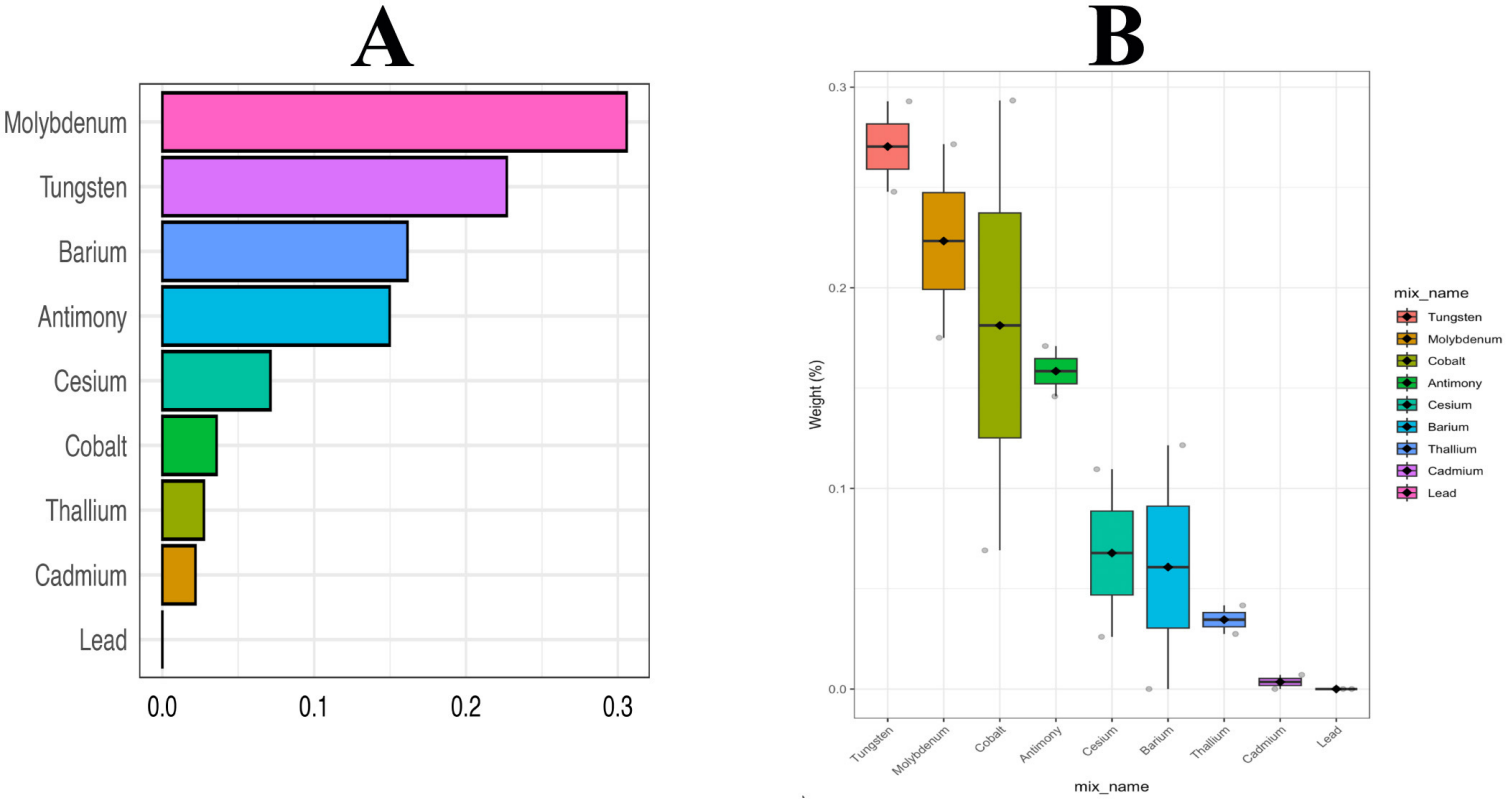
Weighted values of urinary metals for NAFLD in WQS models. Models were adjusted for gender, age, race, education, PIR, marital status, BMI, MET, drinking alcohol status, smoking status, and CCI.

**Figure 4:**
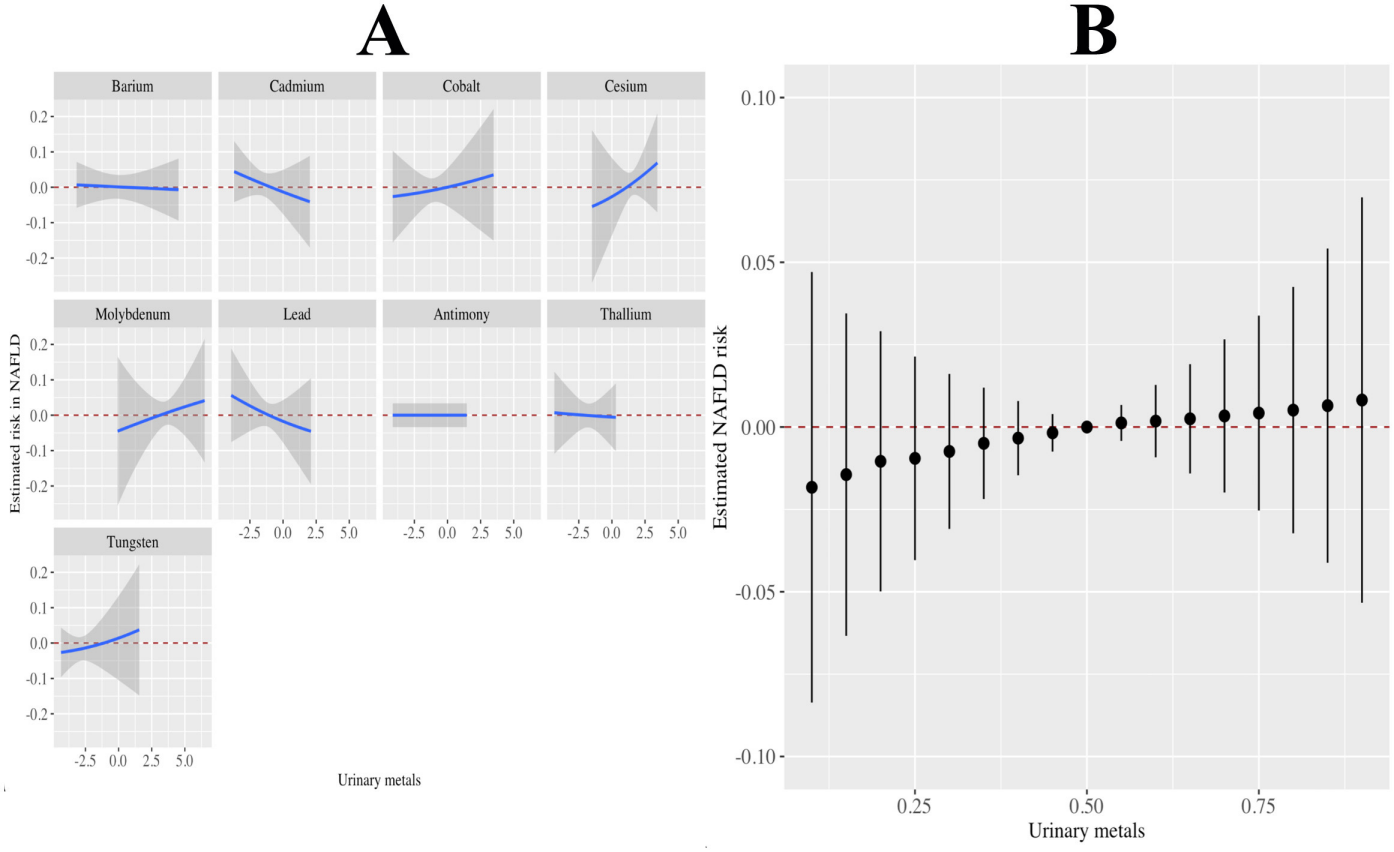
Associations of the urinary metals with NAFLD risk estimated by Bayesian Kernel Machine Regression (BKMR). **(A)** Exposure-response functions for each metals with the other metals fixed at the median. **(B)** Combined effects of urinary metals mixture on NAFLD risk. This plot showed the estimated difference in NAFLD risk and 95% confidence interval when all metals concentrations were held at particular percentiles compared to their medians. Models were adjusted for gender, age, race, education, PIR, marital status, BMI, MET, drinking alcohol status, smoking status, and CCI.

Figure 2 shows the association between LN-transformed metal concentrations and NAFLD risk by weighted multiple logistic regression models. Potential confounders were adjusted for age, sex, PIR, education, marital status, BMI, alcohol consumption, MET, CCI, and smoking status. The highest exposure quantile of Ba (OR: 1.48, 95% CI: 1.10 to 1.99), Cs (OR: 1.38, 95% CI: 0.94 to 2.01), Mo (OR: 1.37, 95% CI: 1.01 to 1.87), Tu (OR: 1.34, 95% CI: 1.00 to 1.80), Tl (OR: 1.29, 95% CI: 0.95 to 1.75), and Cd (OR: 1.29, 95% CI: 0.94to 1.77) increased the risk of NAFLD compared to quantile 1 (all *P* for trend < 0.05). Meanwhile, mixed metals were positively associated with NAFLD risk (OR: 1.48, 95% CI: 1.06 to 2.06) (Figure 2). In addition, the metals with the highest weights in the WQS model were Mo (30.60%), Tu (22.70%), Ba (16.10%), and Sb (15.00%) (Figure 3). Finally, analysis by BKMR model showed that mixed metals were significantly positively associated with NAFLD risk (Figure 4).

### Associations between metal concentration and inflammatory and metabolic markers

**Figure 5:**
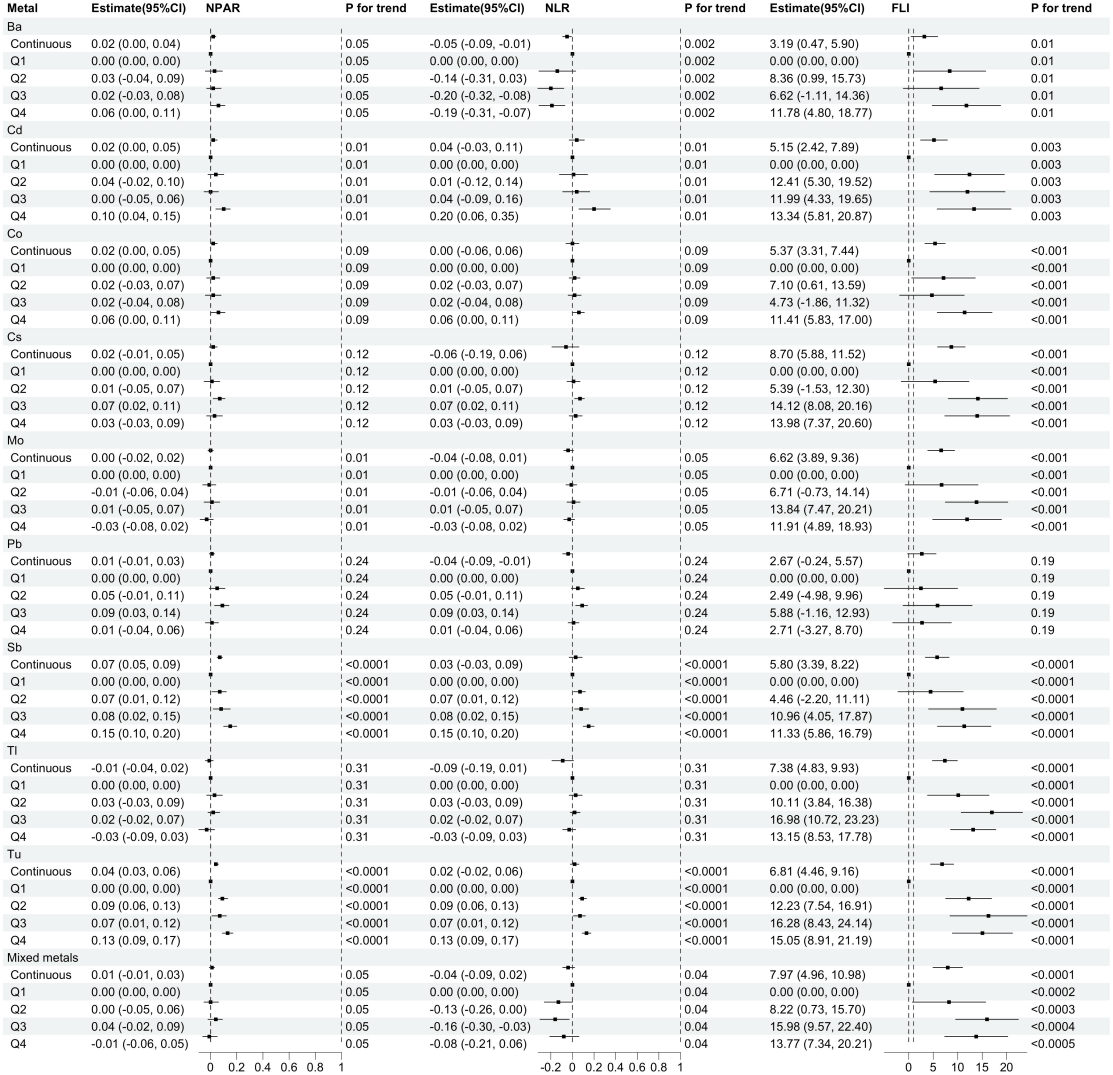
Regression coefficients (95% CI) for inflammatory and metabolic markers associated with single and mixed metal levels. Models were adjusted for gender, age, race, education, PIR, marital status, BMI, MET, drinking alcohol status, smoking status and CCI. Continuous, Ln-transformed concentration of metals; Q, quartile.

Figure 5 shows the association of urinary metals with inflammatory metabolic markers based on linear regression. We found that the highest quartile of Mo, Tu, Ba, Sb, and Cd (compared to quantile 1) was associated with a reduction in NPAR (all *P* for trend < 0.05). With the increase of Mo, Tu, Ba, Sb, and Cd quantiles, NLR also decreased (all *P* for trend < 0.05). Mo, Tu, Ba, Sb, Cd, Co, Cs, and positive correlation between the FLI agreed and Tl (all *P* for trend < 0.05). In addition, mixed metals were negatively associated with NPAR (β: −0.01, 95% CI: −0.06 to 0.05) and NLR (β: −0.08, 95% CI: −0.21 to 0.06), and positively associated with FLI (β: 13.77, 95% CI: 7.34 to 20.21).

### Associations between inflammatory and metabolic markers and NAFLD risk

Supplementary Table 2 shows the associations of inflammatory and metabolic markers with NAFLD risk based on logistic regression. Each unit increase in NPAR was associated with a 94% reduction in NAFLD risk (OR: 0.06, 95% CI: 0.01 to 0.11). Similarly, each 1-unit increase in NLR was associated with a 6% reduction in NAFLD risk (OR: 0.94, 95% CI: 0.87 to 1.01). In addition, each unit increase in FLI was associated with a 1% increase in the OR for NAFLD (OR: 1.01, 95% CI: 1.01 to 1.02), which was consistent with the quantile analysis (Q4 vs. Q1: OR 3.45, 95% CI: 2.11 to 5.63).

### Mediation analyses

To further elucidate the relationship between metal exposure and NAFLD risk, parallel mediation analyses were conducted, focusing on the potential mediating roles of inflammatory and metabolic markers. We found that the NPAR significantly mediates the association of Cd, Co, Mo, Tl, and Tu with NAFLD risk, with mediation percentages of 2.56%, 14.29%, 50.00%, 11.36%, and 66.67%, respectively (*P* < 0.05). Similarly, FLI was identified as a mediator in the connection between NAFLD risk and the metals Ba and Pb, with mediation proportions of 17.07% and 19.40%, respectively, as detailed in Table 2. Furthermore, the study observed that NPAR and NLR, in conjunction with FLI, mediate the association between mixed metal exposure and NAFLD risk. The respective mediation ratios for these markers were 48.36%, 20.28%, and 57.44% (*P* < 0.05), as illustrated in Supplementary Figure 2.

**Table 2:** Inflammatory and metabolic risk markers as mediators in the relationship between single urinary metal levels and NAFLD risk.

**Metals**	**NPAR**	***P* value**	**NLR**	***P* value**	**FLI**	***P* value**
	**Estimation% (95% CI)**		**Estimation% (95% CI)**		**Estimation% (95% CI)**	
**Ba**						
Total effect	−0.008 (−0.026, 0.010)	0.37	−0.009 (−0.025, 0.010)	0.31	0.041 (0.017, 0.070)	0.004
Indirect effect	0.001 (−0.001, 0)	0.18	0.001 (0, 0.002)	0.33	0.007 (0.001, 0.010)	0.016
Direct effect	−0.009 (−0.026, 0.010)	0.32	−0.010 (−0.026, 0.010)	0.31	0.034 (0.009, 0.060)	0.008
**Cd**						
Total effect	0.039 (0.018, 0.060)	<0.001	0.040 (0.020, 0.060)	<0.001	0.006 (−0.022, 0.040)	0.70
Indirect effect	0.001 (−0.001, 0)	0.012	−0.001 (−0.003, 0)	0.28	0.001 (−0.005, 0.010)	0.81
Direct effect	0.038 (0.017, 0.060)	<0.001	0.041 (0.021, 0.060)	<0.001	0.005 (−0.021, 0.040)	0.75
**Co**						
Total effect	0.014 (−0.007, 0.040)	0.22	0.014 (−0.008, 0.040)	0.22	0.015 (−0.017, 0.050)	0.40
Indirect effect	0.002 (−0.001, 0)	<0.001	−0.001 (−0.001, 0)	0.36	−0.007 (−0.014, 0)	0.06
Direct effect	0.012 (−0.009, 0.030)	0.30	0.015 (−0.008, 0.040)	0.22	0.022 (−0.009, 0.050)	0.19
**Cs**						
Total effect	0.053 (0.009, 0.090)	0.028	0.053 (0.008, 0.090)	0.016	0.003 (−0.080, 0.070)	0.87
Indirect effect	0.002 (−0.001, 0)	0.116	0 (−0.001, 0)	0.804	−0.002 (−0.018, 0.010)	0.79
Direct effect	0.051 (0.007, 0.090)	0.028	0.053 (0.008, 0.090)	0.02	0.005 (−0.076, 0.070)	0.83
**Mo**						
Total effect	0.018 (−0.097, 0.050)	0.50	0.021 (−0.091, 0.050)	0.45	−0.056 (−0.104, 0.050)	0.27
Indirect effect	−0.009 (−0.023, 0)	0.004	0.004 (−0.004, 0.020)	0.25	−0.002 (−0.046, 0.040)	0.76
Direct effect	0.027 (−0.083, 0.060)	0.412	0.016 (−0.099, 0.050)	0.50	−0.054 (−0.120, 0.060)	0.29
**Pb**						
Total effect	−0.004 (−0.027, 0.020)	0.716	−0.004 (−0.026, 0.020)	0.73	−0.067 (−0.085, −0.040)	<0.001
Indirect effect	−0.003 (−0.006, 0)	0.004	0.001 (−0.001, 0)	0.32	−0.013 (−0.020, −0.010)	<0.001
Direct effect	−0.001 (−0.025, 0.030)	0.92	−0.004 (−0.027, 0.020)	0.70	−0.054 (−0.074, −0.030)	<0.001
**Sb**						
Total effect	−0.020 (−0.034, 0)	0.032	−0.197 (−0.337, 0)	0.024	−0.019 (−0.039, 0.020)	0.20
Indirect effect	0.001 (0, 0.001)	0.008	−0.020 (−0.040, 0)	0.896	0.004 (−0.001, 0.010)	0.15
Direct effect	−0.021 (−0.035, 0)	0.02	−0.177 (−0.336, 0)	0.024	−0.023 (−0.042, 0.010)	0.11
**Tl**						
Total effect	−0.044 (−0.060, −0.020)	<0.001	−0.045 (−0.061, −0.020)	0.004	−0.004 (−0.044, 0.050)	0.82
Indirect effect	−0.005 (−0.008, 0)	<0.001	0.001 (0, 0.002)	0.296	0.007 (−0.004, 0.020)	0.21
Direct effect	−0.039 (−0.056, −0.020)	0.004	−0.046 (−0.061, −0.020)	0.004	−0.011 (−0.051, 0.040)	0.59
**Tu**						
Total effect	−0.003 (−0.021, 0.020)	0.74	−0.003 (−0.021, 0.020)	0.80	0.009 (−0.019, 0.040)	0.62
Indirect effect	0.002 (0.001, 0.003)	0.004	−0.001 (−0.001, 0)	0.52	0.010 (0.003, 0.020)	<0.001
Direct effect	−0.005 (−0.023, 0.010)	0.58	−0.002 (−0.021, 0.020)	0.82	−0.001 (−0.028, 0.030)	0.94

### Non-linearity analysis using RCS

To investigate the association between blood metals and NAFLD incidence, we carefully investigated the non-linear relationship between metals and NAFLD incidence probability by smooth curve fitting using the penalty spline method (Supplementary Figure 3). We performed analyses using constrained spline models and found that each metal was positively associated with NAFLD incidence even after adjusting for potential confounders such as age, sex, race, education level, marital status, household income, body mass index, smoking status, alcohol consumption, and comorbidity index. RCS showed that with the increase of Ba, Cs, and Pb concentrations, the probability of NAFLD increased, and the change rate was first slow and then accelerated. With the increase of Cd, Mo, Sb, and Tu concentrations, the probability of NAFLD increased, and then gradually tended to be stable. Co and Tl with the increase of concentration, NAFLD probability increased.

## Discussion

This study examined the association between nine urinary metals and NAFLD risk among 3,948 U.S. adults. We observed that Mo, Tu, Ba, Cd, Cs, Tl, and their mixture were positively associated with an increased likelihood of NAFLD. Further analysis indicated that the metals negatively correlated with inflammatory markers (NPAR and NLR) but positively correlated with metabolic markers (FLI). These inflammatory and metabolic markers mediated 16.89%−69.39% of the association between metals and NAFLD risk. Our findings highlight the role of metals in NAFLD etiology potentially through influencing inflammation and metabolism. Recent findings align with our results, suggesting that heavy metal exposures contribute to NAFLD risk by affecting metabolic and inflammatory pathways. For instance, Lee et al. (13) identified similar associations in Korean adolescents, emphasizing the global relevance of metal-associated NAFLD. Additionally, Xie et al. (14) highlighted the role of metal mixtures in metabolic-associated fatty liver disease using NHANES data. Apart from that, recent research by Li et al. (27) demonstrated that urinary heavy metal mixtures, such as cadmium and molybdenum, are associated with high remnant cholesterol levels, a metabolic marker closely related to NAFLD risk.

Several possible mechanisms may explain the observed associations of metals with increased NAFLD risk. First, certain metals can induce oxidative stress and impair mitochondrial function. In particular, Mo can elevate reactive oxygen species (ROS) generation and reduce glutathione levels, triggering oxidative injury (28). Cd also promotes lipid peroxidation and interferes with endogenous antioxidants (29). Oxidative stress damages cell membranes, proteins, lipids, and DNA, eventually causing hepatocyte death and liver dysfunction (30). Sodium (Na) intake has also been linked to NAFLD. High-sodium diets increase oxidative stress and lipogenesis, while severe sodium restriction can impair metabolic health, suggesting a complex relationship with NAFLD (31). Second, metals like Cs and Tl disrupt potassium homeostasis in hepatocytes, depolarizing the mitochondrial membrane potential and activating apoptotic signaling cascades (32). Loss of potassium gradients across the mitochondrial membrane represents an early event in cell death. Third, metals may impair lipid metabolism by upregulating lipogenic genes or downregulating enzymes regulating fatty acid oxidation (33). For instance, chronic low-level Cd exposure stimulates de novo lipogenesis by activating the sterol regulatory element-binding protein-1c (SREBP-1c) and carbohydrate response element binding protein (ChREBP) (34). Alterations in hepatic lipid metabolism can facilitate excessive lipid accumulation within hepatocytes. Iron (Fe) is another key factor in NAFLD. Excess hepatic iron promotes oxidative stress and ferroptosis, contributing to lipid peroxidation and liver damage (35). Fourth, certain metals such as Ba and Cs compete with essential ions like potassium and calcium for binding sites, potentially interfering with metal-dependent enzymes and biological processes (36, 37). Fifth, metals can elicit endocrine disruption by mimicking or blocking hormone actions, disrupting hormonal feedback loops (38). For example, Cd mimics estrogen signaling and may promote NAFLD partly through this endocrine-disrupting mechanism (39). Overall, metals likely contribute to NAFLD through oxidative damage, cell death, metabolic dysregulation, ionic imbalances, and endocrine disruption.

Notably, we discovered associations between metals and reductions in two inflammatory markers—NPAR and NLR. On the other hand, metals positively correlated with the metabolic fatty liver index. Although unexpected, these findings align with emerging research indicating bidirectional crosstalk between inflammation and metabolism in NAFLD pathogenesis. Obesity can trigger chronic low-grade inflammation mediated through nutritional excess and adipocyte dysfunction (40). Additionally, occupational exposure may play a critical role in high-risk groups, as certain industries, such as mining, welding, and battery production, involve significant exposure to metals like lead, cadmium, and mercury (41). Workers in these industries may experience elevated levels of metals due to prolonged and direct contact, underscoring the importance of targeted preventive measures and occupational health policies (42). Early in NAFLD, oxidative stress stimulates inflammatory pathways such as c-Jun N-terminal kinase (JNK) and IkappaB kinase beta (IKKβ)/nuclear factor kappa-light-chain-enhancer of activated B cells (NF-κB) signaling cascades. Subsequently, inflammatory cytokines like tumor necrosis factor alpha (TNFα), interleukin 6 (IL6), and interleukin 1 beta (IL1β) exacerbate insulin resistance and metabolic dysfunction (43). However, during later stages, excessive hepatocyte death and fibrosis may limit local inflammatory responses (44). The FLI incorporates anthropometric and laboratory parameters related to metabolism and can detect worsening metabolic deregulation even amid dampening inflammation. Therefore, declining NPAR/NLR levels alongside rising FLI scores likely signify disease progression from simple steatosis to advanced fibrosis/cirrhosis.

The mediation analyses provide further insight into potential mechanisms. The associations between metals and NAFLD may occur partly through inflammation and metabolic pathways. Mo, Tu, Cd, Co, and Tl mediated NAFLD risk via NPAR, while Ba and Pb mediated risk through FLI. Consistently, the mixed metals' relationship with NAFLD was mediated by all three markers. These findings agree with *in vitro* experiments demonstrating that certain metals trigger inflammatory signaling and metabolic alterations that promote hepatocellular lipid accumulation. For example, Co elevates TNFα, IL6, IL1β and toll-like receptor 4 (TLR4) levels in hepatocytes (44). Mo also causes TNFα overexpression and represses peroxisome proliferator-activated receptor alpha (PPARα) activity (45). Furthermore, in animal models, Pb impairs lipid homeostasis genes like PPARα, inducing hepatic steatosis (46). Therefore, metals may elicit inflammatory reactions and disturb metabolic regulation early during NAFLD pathogenesis. But with progressive inflammation and ballooning degeneration of hepatocytes, declining inflammatory activity gives way to worsening metabolic dysfunction (24). Hence, inflammation and metabolism likely contribute jointly yet sequentially to NAFLD induced by chronic metal exposures.

More strikingly, Mo, Tu, Ba, and Sb were top contributors in the WQS model, highlighting their importance. These subtler exposures are easily overlooked compared to better-known hepatotoxic metals like arsenic or mercury (47, 48). However, our WQS analysis indicates these underappreciated metals significantly impact NAFLD risk. Occupational settings involving steel production, mining, metal smelting and glass manufacturing may confer Mo/Tu exposures (49, 50). Ba additionally occurs near drilling sites and antimony trioxide is utilized as a flame retardant (51). While such exposures appear modest individually, their synergistic adverse effects likely drive NAFLD risk substantially. Nevertheless, arsenic, mercury and other hepatotoxic metals still warrant examination regarding NAFLD despite their exclusion presently due to insufficient detection rates. Future investigations must characterize the relative toxicity of different metals in NAFLD development.

Some study limitations should be acknowledged. First, the cross-sectional nature prevents causal determinations regarding temporality. However, animal and *in vitro* studies support that metals trigger oxidative stress, inflammation and metabolic changes that initiate hepatic lipid accumulation. Second, urinary metals indicate only recent exposures rather than long-term bioaccumulation. Still, they provide reasonable surrogates for bodily metal burden. Third, fatty liver assessments through vibration-controlled transient elastography lack histological confirmation. Non-etheless, this approach demonstrates excellent diagnostic accuracy for steatosis vs. advanced fibrosis/cirrhosis (52). Fourth, residual confounding from unmeasured factors cannot be fully excluded. Yet, we adjusted extensively for likely demographic, socioeconomic, lifestyle and medical confounders of NAFLD risk. Fifth, although nationally-representative, generalizability to other populations with differing genetic backgrounds or environmental metal levels may be limited. Still, these data likely reflect exposures among U.S. adults accurately. Finally, this study did not include sodium and iron in its analysis, as these elements were outside the scope of our predefined objectives, which focused on the potential roles of urinary heavy metals in NAFLD risk. Future studies could explore the roles of sodium and iron to provide a more comprehensive understanding of their potential contributions to NAFLD.

In this study, we conducted a comprehensive analysis of the relationship between nine urinary metals and the risk of NAFLD in a large, nationally representative sample of the U.S. population. Our findings reveal a notable link, where various metals appear to increase the likelihood of NAFLD, potentially through inducing inflammation and metabolic disturbances. This research underscores the impact of harmful environmental exposures, contributing to the escalating global prevalence of NAFLD. Considering projections that NAFLD might become the predominant reason for liver transplants by 2030, addressing heavy metal exposures and other risk factors emerges as a critical public health imperative. Future research should focus on elaborating the mechanisms and specific toxicities of these metals in the context of NAFLD. Longitudinal cohort studies are also crucial for establishing a clear temporal relationship between metal exposure and the onset and progression of NAFLD. Ultimately, identifying these modifiable risk factors and implementing effective prevention strategies will be key to combating the global NAFLD epidemic.

## Conclusions

In conclusion, our results show that the single and mixed metal is positively correlated with an increased risk of NAFLD, this is mainly driven by Mo, Tu, and Ba. In addition, we found that the metal exposure is associated with inflammation and metabolic markers, and these markers associated with risk of NAFLD. Inflammation and metabolic dysfunction have been identified as critical mediators in the progression of NAFLD (18, 21). Recent studies have also demonstrated similar findings in different populations, suggesting that heavy metal exposure contributes to NAFLD risk through metabolic and inflammatory pathways (1, 14). In addition, the mediation analysis shows that metal associated with NAFLD risk may be mediated by inflammation and metabolic markers. These findings identify the risk factors of NAFLD and suggests that inflammation and metabolic markers is metal the potential mechanism of an adverse effect on fatty liver disease. Addressing environmental exposures and improving metabolic health are crucial for mitigating the rising global prevalence of NAFLD (7, 53).

## Data Availability

The original contributions presented in the study are included in the article/Supplementary material, further inquiries can be directed to the corresponding author.
