# Supplementary material for: Inflammatory and metabolic markers mediate the association between urinary metals and non-alcoholic fatty liver disease in U.S. adults: a cross-sectional study
Source: Front Public Health. 2025 Jul 4;13:1564302. doi: 10.3389/fpubh.2025.1564302 (PMC12271117; doi:10.3389/fpubh.2025.1564302)
Supplement: Supplementary file 1 [file Table_1.docx]

Supplementary Material

Figure S1. Pearson’s correlation matrix among Ln-transformed urinary metals in the study population.

Figure S2. Estimated proportion of the association between urinary mixed metals and NAFLD mediated by NPAR (A), NLR (B), and FLI (C). Models were adjusted for gender, age, race, education, PIR, marital status, BMI, MET, drinking alcohol status, smoking status and CCI. IE, the estimate of the indirect effect; DE, the estimate of the direct effect; Proportion of mediation = IE/DE + IE.

Figure S3. The non-linear relationship between Ln-transformed urinary metals and NAFLD.

Table S1. Distributions of metals in the study population.

Table S2. Associations of inflammatory and metabolic risk markers with NAFLD risk.

Table S1. Distributions of metals in the study population.

| Metals (μg/L) | Detection rate (%) | Median | Interquartile range |
| --- | --- | --- | --- |
| Ba | 100.00 | 0.95 | 0.44-1.95 |
| Cd | 100.00 | 0.23 | 0.11-0.44 |
| Co | 100.00 | 0.36 | 0.19-0.59 |
| Cs | 100.00 | 4.60 | 2.74-6.80 |
| Mo | 100.00 | 35.73 | 18.40-62.90 |
| Pb | 100.00 | 0.32 | 0.18-0.57 |
| Sb | 100.00 | 0.04 | 0.03-0.08 |
| Tl | 100.00 | 0.17 | 0.10-0.26 |
| Tu | 100.00 | 0.06 | 0.03-0.11 |

| Table S2. Associations of inflammatory and metabolic risk markers with NAFLD risk. | | | | | | |
| --- | --- | --- | --- | --- | --- | --- |
| Marks | Continuous | Q1 | Q2 | Q3 | Q4 | *P* for trend |
|  | OR (95% CI) |  | OR (95% CI) | OR (95% CI) | OR (95% CI) |  |
| NPAR | 0.06(0.01,0.11) | 1.00 (reference) | 0.87(0.64,1.20) | 0.86 (0.69,1.06) | 0.97 (0.78,1.21) | 0.01 |
| NLR | 0.94 (0.87, 1.01) | 1.00 (reference) | 1.14(0.60,2.16) | 0.80(0.44,1.43) | 0.60(0.28,1.27) | 0.05 |
| FLI | 1.01 (1.01, 1.02) | 1.00 (reference) | 3.19(2.07,4.92) | 2.85(1.55,5.26) | 3.45(2.11,5.63) | <0.0001 |
| Models were adjusted for age, gender, race, education, PIR, marital status, BMI, MET, drinking alcohol status, smoking status and CCI. | | | | | | |


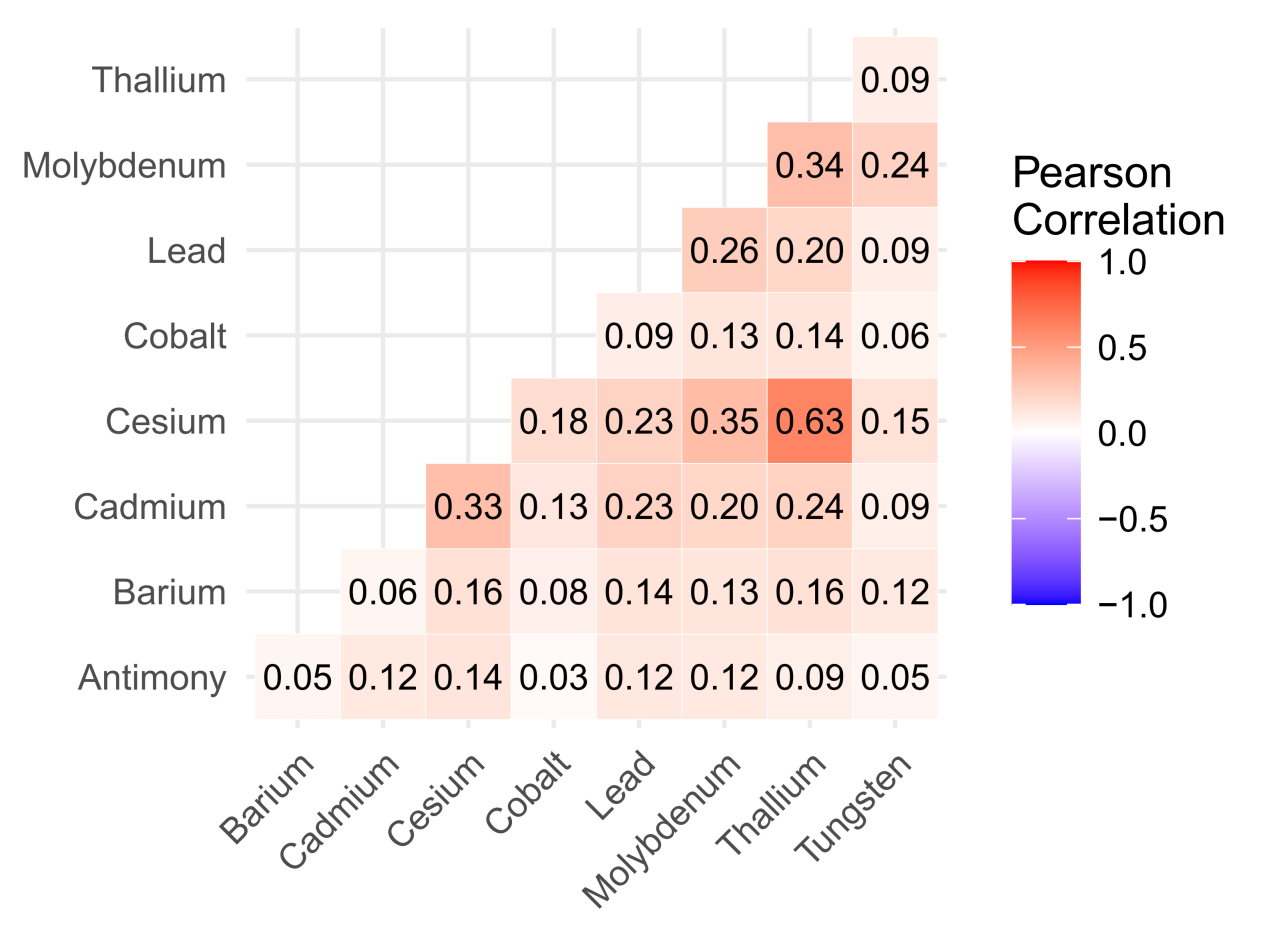


Figure S1. Pearson’s correlation matrix among Ln-transformed urinary metals in the study population.


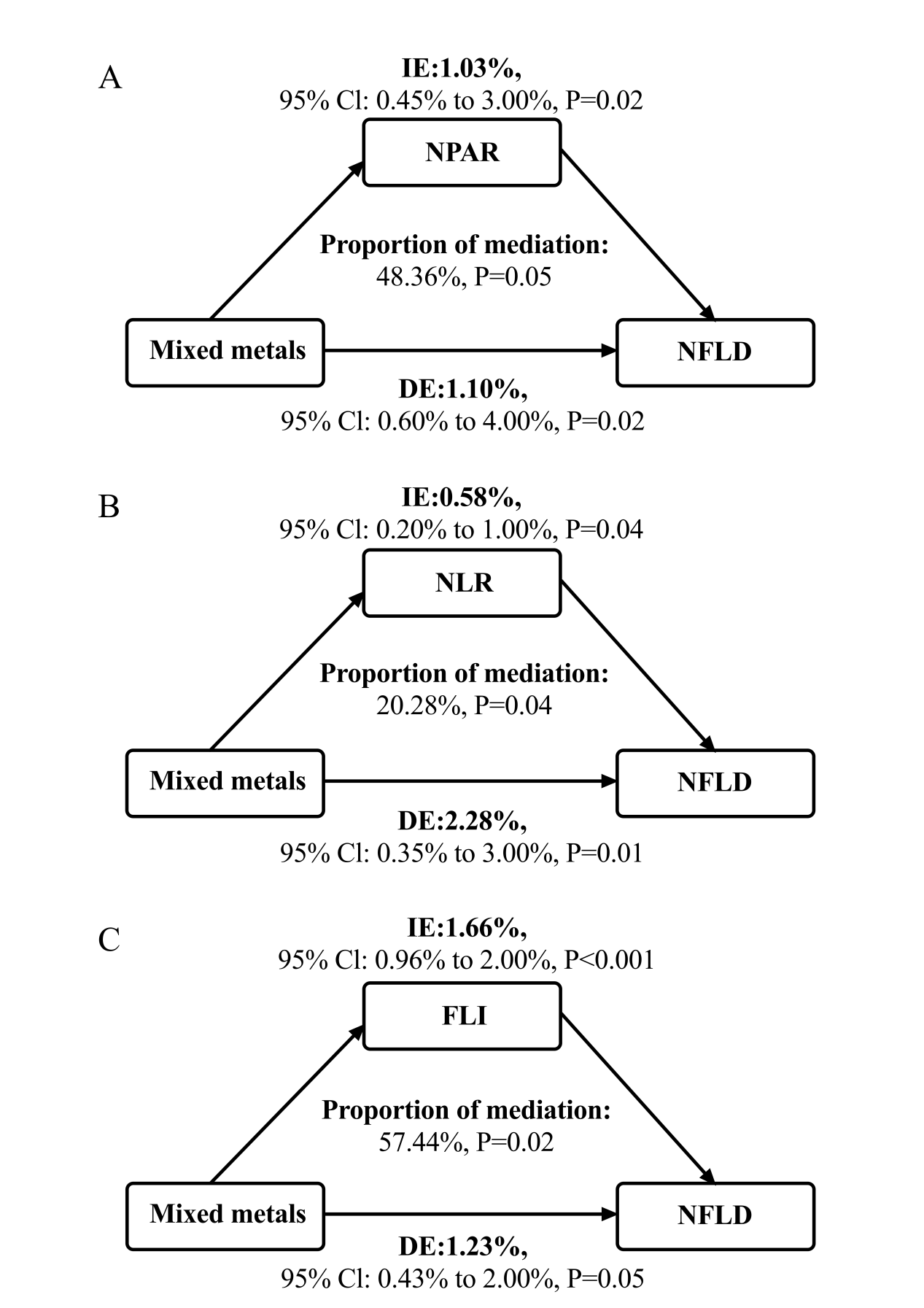


Figure S2. Estimated proportion of the association between urinary mixed metals and NAFLD mediated by NPAR (A), NLR (B), and FLI (C). Models were adjusted for gender, age, race, education, PIR, marital status, BMI, MET, drinking alcohol status, smoking status and CCI. IE, the estimate of the indirect effect; DE, the estimate of the direct effect; Proportion of mediation = IE/DE + IE.


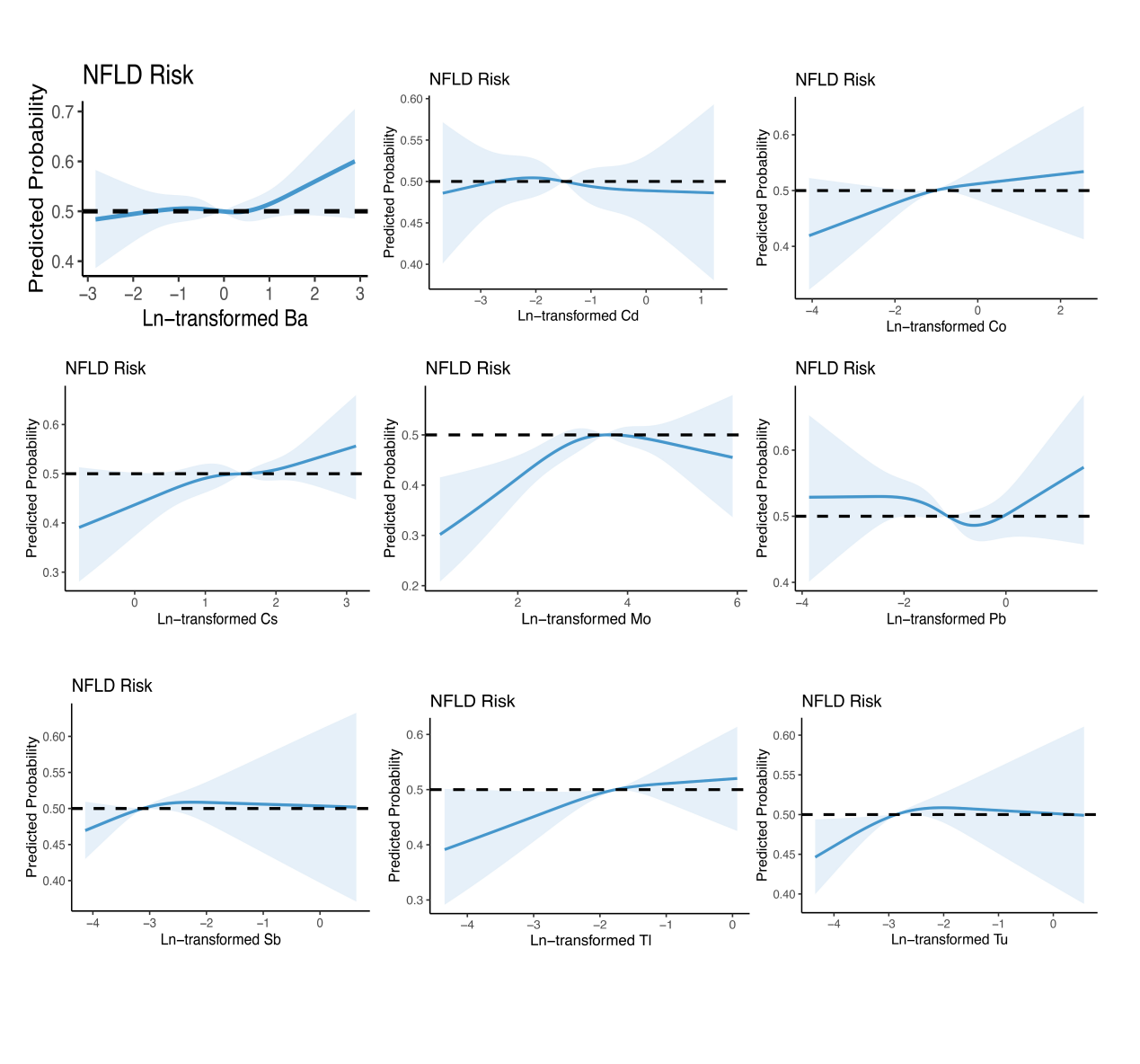


Figure S3. The non-linear relationship between Ln-transformed urinary metals and NAFLD.
